# Prognostic Significance of Biventricular and Biatrial Strain in Dilated Cardiomyopathy: Strain Analysis Derived from Cardiovascular Magnetic Resonance

**DOI:** 10.31083/j.rcm2412347

**Published:** 2023-12-12

**Authors:** Shengliang Liu, Yunling Li, Jianxiu Lian, Xueying Wang, Ye Li, Di Wang, Yanming Zhao, Zhiyuan Wu, Xia Gu, Bing Xu, Jinjin Cui, Xuedong Wang, Jiayue Ren, Qiang Li, Guokun Wang, Bo Yu

**Affiliations:** ^1^Cardiovascular Imaging Center, Department of Cardiology, The Second Affiliated Hospital of Harbin Medical University, 150086 Harbin, Heilongjiang, China; ^2^Department of Biomedical Engineering, Beihang University, 100191 Beijing, China; ^3^Department of Cardiology, The Second Affiliated Hospital of Harbin Medical University; The Key Laboratory of Myocardial Ischemia, Chinese Ministry of Education,150086 Harbin, Heilongjiang, China; ^4^Department of Vascular Surgery, Beijing Hospital, National Center of Gerontology; Institute of Geriatric Medicine, Chinese Academy of Medical Sciences, 100730 Beijing, China

**Keywords:** dilated cardiomyopathy, cardiovascular magnetic resonance, global radial strain, major adverse cardiac event

## Abstract

**Background:**

Dilated cardiomyopathy (DCM) has a poor prognosis and high mortality. The relationship between the deformation capacity of the biatrial and biventricular regions in patients with DCM remains unclear.

**Methods:**

This retrospective study used cardiovascular magnetic resonance (CMR) to assess patient enrollment between September 2020 to May 2022. Feature tracking (FT) was used to evaluate biventricular global radial strain (GRS), global circumferential strain (GCS) and global longitudinal strain (GLS). Fast long-axis method was used to evaluate biatrial GLS by analyzing balanced steady-state free precession cine images. The median follow-up period was 362 days (interquartile range: 234 to 500 days). DCM patients were divided into two groups based on the occurrence or non-occurrence of major adverse cardiac event (MACE). The primary endpoint was defined as all-cause death, heart transplantation, and adverse ventricular arrhythmia. The secondary end point included hospitalizations due to heart failure. Cox regression analysis was utilized for variables and Kaplan-Meier survival was utilized for clinical outcomes.

**Results:**

There were 124 DCM patients (52.82 ± 12.59 years, 67.74% male) and 53 healthy volunteers (53.17 ± 14.67 years, 52.83% male) recruited in this study. Biventricular GRS, GCS, GLS, and biatrial GLS were significantly impaired in the DCM group compared with the healthy group. In receiver-operating characteristic curve, biatrial GLS and biventricular GRS, GCS, and GLS showed significant prognostic value in predicting MACEs (all *p* < 0.05). In multivariate Cox regression analysis, left ventricular (LV) GLS offered a significant and independent prognostic value surpassing other CMR parameters in predicting MACE. In Kaplan-Meier analysis, patients with a LV GLS >–4.81% had a significantly higher rate of MACE (Log-rank *p* < 0.001).

**Conclusions:**

LV GLS was independently associated with MACEs in DCM patients by using FT and fast long-axis method derived from CMR. Comprehensive CMR examination including biatrial and biventricular functions should be systematically performed, to understand disease characteristics, as well as improve the risk stratification and therapeutic management for patients with DCM.

## 1. Introduction

Dilated cardiomyopathy (DCM) remains a serious medical condition, and the challenges associated with risk stratification in DCM continue to pose ongoing challenges in clinical practice [1, 2]. Currently, cardiovascular magnetic resonance (CMR) is considered the gold standard for evaluating cardiac morphology, function, and tissue characterization [3]. Left ventricular (LV) ejection fraction (LVEF) has been the main criteria for evaluation of therapy in DCM [4]. However, studies have shown that improvement in LVEF following systemic therapy does not necessarily indicate recovery of systolic function [5, 6]. Furthermore, it has been established that left atrial (LA) volume and booster function are independent predictors of outcomes in DCM [7, 8], as atrial function is tightly coupled to ventricular relaxation and diastolic properties [9]. Studies have demonstrated that LA strain is a more sensitive measure than LA morphologic and functional alterations when reflecting LV diastolic dysfunction [10, 11].

Recent studies have highlighted the increasing potential of CMR in assessing myocardial deformation and its ability to predict clinical outcomes, surpassing traditional parameters [12, 13, 14]. Feature tracking (FT) has emerged as a useful technique for identifying subtle ventricular systolic dysfunction and calculating myocardial strain, exhibiting favorable consistency with echocardiography and other CMR techniques [15, 16, 17]. For atrial myocardial deformation, the fast long-axis method has shown superior stability, reliability, and reproducibility when compared to the FT method for obviating LA appendage and pulmonary veins [18, 19]. Moreover, LV global longitudinal strain (GLS), right ventricular (RV) GLS, and LA conduit strain showed significant prognostic value in individuals with DCM [20, 21, 22].

A comprehensive analysis of biatrial and biventricular myocardial deformation in DCM may contribute to improve risk stratification and implement treatment guidance. The aim of this study was to evaluate prognostic value in DCM patients by analyzing biventricular global radial strain (GRS), global circumferential strain (GCS), and GLS through FT, as well as biatrial GLS through fast long-axis method.

## 2. Materials and Methods

### 2.1 Study Subjects

We retrospectively and consecutively enrolled participants who underwent CMR from September 2020 to May 2022. Two cohorts were recruited: (i) patients with non-ischemic DCM, which were defined as impaired systolic function with LVEF ≤45% and dilated LV end-diastolic diameter (EDD) measured by echocardiography according to the latest European Society of Cardiology proposal [23], and (ii) healthy volunteers who were free of any history of medical conditions. Exclusion criteria included the following: subjects with a previous history of myocardial infarction or significant coronary artery disease (stenosis >50%) determined by coronary angiography; primary valvular disease (severe mitral regurgitation, moderate and severe aortic regurgitations, or aortic stenosis <1 ${\mathrm{cm}}^{2}$); hypertensive or congenital heart disease; acute myocarditis; and diagnosis of arrhythmogenic cardiomyopathy [24]. Patients with atrial fibrillation were excluded as an irregular heart rhythm affects image quality and strain measurement.

### 2.2 Image Acquisition

CMR imaging was performed with a 3.0-Tesla scanner (Ingenia CX, Philips Healthcare, the Netherlands) using a 32-channel phased-array abdomen coil. Cine images were performed by using a steady-state free procession (SSFP) sequence with multiple breath holds and electrocardiographic gating. The scanning parameters were as follows: field of view (FOV) = 300 × 300 ${\mathrm{mm}}^{2}$, repetition time (TR)/echo time (TE) = 2.8/1.42 ms, flip angle = 45°, voxel = 1.8 × 1.6 × 8.0 mm, and 8-mm slice thickness. Late gadolinium enhancement (LGE) images were performed 10 to 15 minutes after intravenous administration of 0.1 mmol/kg of gadolinium-based contrast agent (Bayer Healthcare, Germany) by using three-dimensional phase sensitive inversion recovery (PSIR) sequence. The scanning parameters were FOV = 300 × 300 ${\mathrm{mm}}^{2}$, TR/TE = 6.1/3.0 ms, flip angle = 25°, voxel = 1.8 × 1.68 × 8.0 mm, and 8-mm slice thickness.

### 2.3 Image Post-Procession

All CMR images were post-analyzed with a commercially available workstation (cvi42, Circle Cardiovascular Imaging Inc., Calgary, Alberta, Canada). The analysis of cardiac function and morphology was based on SSFP cine images, biventricular endocardial and epicardial borders, biatrial atrioventricular junctions, and midpoints of the posterior atrial wall. These measurements were tracked automatically based on manual calibration performed by two radiologists (each with 3 or 4 years of experience in CMR) who were blinded to baseline and outcome data. The produced measurements of left and right ventricular ejection fraction (RVEF), cardiac output (CO), end-diastolic volume (EDV), end-systolic volume (ESV), and atrial volumetric calculations. Atrial empty fraction (EF) was obtained according to the volumetric measurements. LV mass was calculated as myocardial gravity (1.05 g/mL) multiplied by total myocardium volume without papillary muscles.

**Fig. 1.**
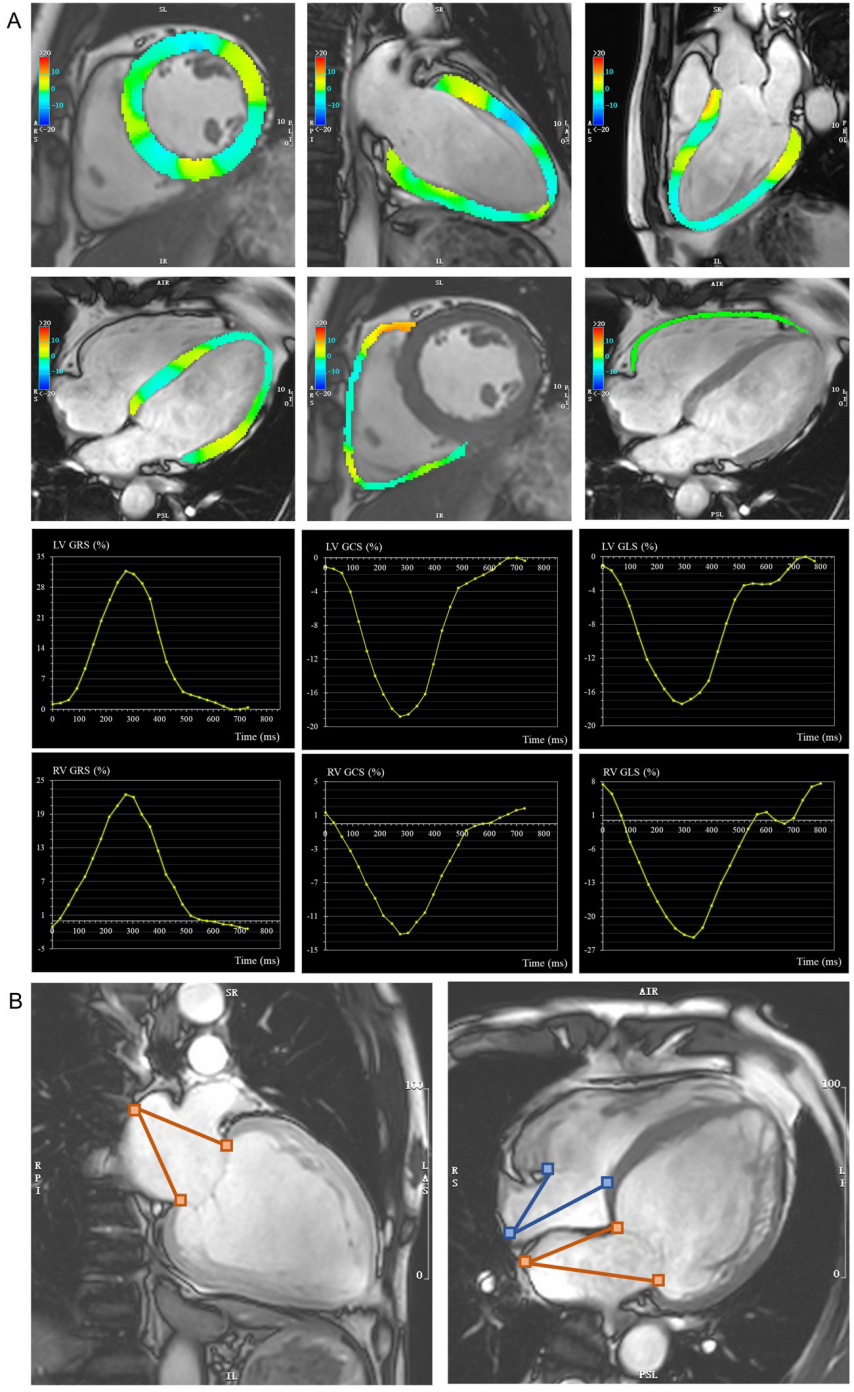
**Representative images of ventricular and atrial strain measurement**. (A) Feature tracking was used for the evaluation of biventricular GRS, GCS and GLS. Radial and circumferential strain were analyzed from short-axis planes, longitudinal strain was analyzed from long-axis planes. (B) Fast long-axis method was used for evaluating biatrial GLS according to the distance between atrioventricular junction and midpoint of posterior atrial wall. GCS, global circumferential strain; GLS, global longitudinal strain; GRS, global radial strain; LV, left ventricular; RV, right ventricular.

CMR-FT derived strain parameters (radial strain, circumferential strain, and longitudinal strain) were used to evaluate ventricular deformation capacity through the use of cine images. Radial strain and circumferential strain were measured via analysis of two-dimensional short-axis planes. Longitudinal strain was measured by analyzing two-dimensional long-axis planes (2-chamber, 3-chamber, and 4-chamber views) (Fig. 1). Atrial strain obtained by fast long-axis method included longitudinal orientation. Atrial longitudinal strain was calculated according to the distance between atrioventricular junction and midpoint of posterior atrial wall (Fig. 1). The peak of the global curve was used as the global strain value. LGE was quantified using mean ± 5 standard deviations algorithm and displayed as a volumetric proportion of the total LV myocardium.

### 2.4 Follow-up Study

Clinical follow-up was performed via structured questionnaires [25] by telephone and then assessed by two experienced cardiologists. The primary endpoints included all-cause death, heart transplantation, and life-threatening arrhythmias. The secondary endpoint was hospitalization due to heart failure. Major adverse cardiac events (MACEs) were included in both primary and secondary endpoints.

### 2.5 Intraobserver and Interobserver Reproducibility

Intra- and interobserver reproducibility for LV GLS were evaluated in 30 randomly selected study subjects. Intraobserver reproducibility was conducted 4 months later by a single radiologist who was blinded to the first analysis results. Interobserver reproducibility was assessed by two experienced radiologists who were blinded, without access to the other’s findings.

### 2.6 Statistics

Normally distributed continuous variables were expressed as mean ± standard deviation and compared by Student’s *t*-test between two groups. Non-normally distributed continuous variables were expressed as medians with interquartile range (IQR) and obtained by Mann-Whitney U test. Categorical variables were shown as numbers with percentage and compared by Fisher’s exact or chi-square test. Univariate and multivariate Cox regression analysis was utilized to verify the prognostic value of CMR parameters for predicting MACE. Pearson or Spearman’s correlation coefficients were used to explore the correlations between myocardial strain and other CMR parameters. Area under the curves (AUCs), specificity, sensitivity and optimal cut-off values, were analyzed by receiver-operating characteristic (ROC) curve. The Kaplan-Meier survival curve was used for survival analysis and compared by log-rank test. Intra- and interobserver variabilities were assessed using intraclass correlation coefficients (ICC). All data were calculated by SPSS 26.0.0 (SPSS Incorporation, Chicago, IL, USA) or MedCalc (version 20, MedCalc Software, Ostend, Belgium). A value of *p* < 0.05 was considered statistically significant.

## 3. Results

### 3.1 Participant Characteristics

**Fig. 2.**
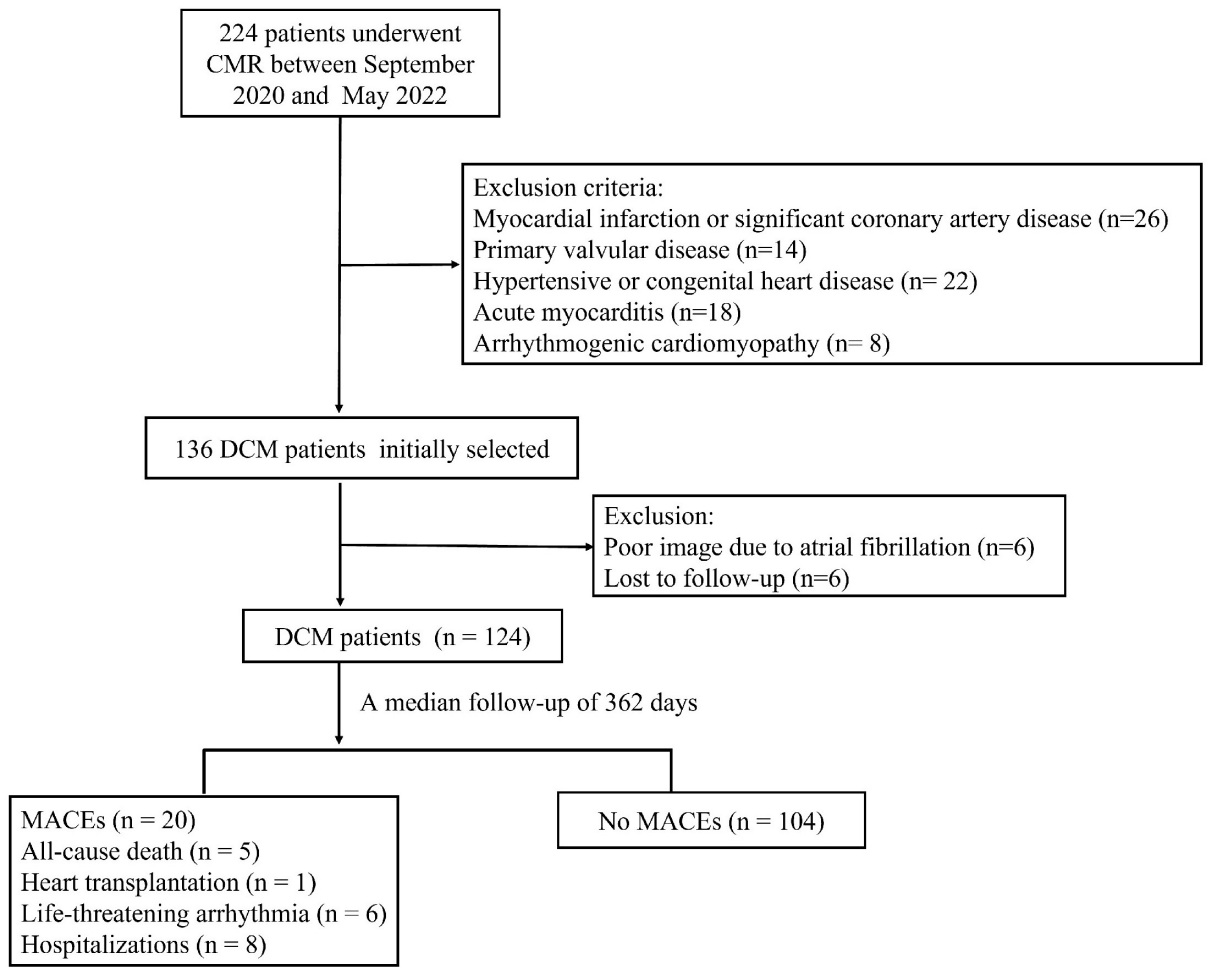
**Inclusion criteria**. This flow chart illustrates the inclusion and exclusion criteria used in the clinical study. Participants progressed through the study based on their eligibility and adherence to specific criteria. CMR, cardiovascular magnetic resonance; DCM, dilated cardiomyopathy; MACE, major adverse cardiac event.

The study consisted of 177 participants, including 124 DCM patients (70%), and 53 healthy volunteers (30%) (Fig. 2). In comparison to healthy volunteers, DCM patients had higher prevalence of hypertension, diabetes mellitus, hyperlipidemia, smoking, left bundle branch block (LBBB), and presented more often with New York Heart Association (NYHA) functional class >II (all *p* < 0.05). Additionally, DCM patients showed higher levels of N-terminal pro-B-type natriuretic peptide (NT-proBNP), troponin I (TnI), creatine kinase myocardial band (CKMB), and were more likely to be receiving angiotensin converting enzyme inhibitor (ACEI) or angiotensin receptor blocker (ARB), beta blockers, diuretics, or digoxin. There were no statistical differences between DCM and healthy group in terms of age, sex, height, and weight (all *p* > 0.05) (Table 1).

**Table 1.** **Baseline characteristics of healthy group, DCM group, no event group, and event group**.

		Healthy (n = 53)	DCM (n = 124)	p*	No event (n = 104)	Event (n = 20)	* $p^{\#}$ *
Age (years)		53.17 ± 14.67	52.82 ± 12.59	0.873	52.66 ± 12.42	53.65 ± 13.74	0.75
Male (n, %)		28 (52.83)	84 (67.74)	0.059	69 (66.35)	15 (75.00)	0.448
Height (cm)		167.91 ± 7.96	167.81 ± 8.5	0.947	167.73 ± 8.42	168.25 ± 9.14	0.804
Weight (kg)		70.75 ± 12.94	71.17 ± 15.93	0.866	71.12 ± 15.32	71.4 ± 19.24	0.943
BSA ($m^{2}$)		1.78 ± 0.2	1.78 ± 0.24	0.901	1.78 ± 0.23	1.79 ± 0.29	0.899
HBP (n, %)		3 (5.66)	27 (21.77)	0.009	24 (23.08)	3 (15.00)	0.561
DM (n, %)		2 (3.77)	27 (21.77)	0.003	21 (20.19)	6 (30.00)	0.377
Hyperlipidemia (n, %)		7 (13.20)	91 (73.39)	<0.001	77 (74.04)	14 (70.00)	0.708
Smoking (n, %)		6 (11.32)	32 (25.80)	0.032	29 (27.88)	3 (15.00)	0.228
LBBB (n, %)		0 (0)	19 (15.32)	0.003	19 (18.27)	0 (0)	0.041
NYHA class				<0.001			<0.001
	I (n, %)	53 (100)	11 (8.87)	-	11 (10.58)	0	-
	II (n, %)	0	24 (19.35)	-	24 (23.08)	0	-
	III (n, %)	0	49 (39.52)	-	39 (37.50)	10 (50)	-
	IV (n, %)	0	40 (32.26)	-	30 (24.19)	10 (50)	-
NT-pro BNP (pg/mL)		0 (0, 118)	5802 (2176, 9179)	<0.001	1798 (887, 5145)	7559 (3983, 12167)	<0.001
TnI (ug/L)		-	0.02 (0, 0.07)	<0.001	0.02 (0, 0.06)	0.04 (0, 0.56)	0.07
CKMB (ug/L)		-	0.80 (0.50, 1.50)	<0.001	0.80 (0.50, 1.40)	1.10 (0.65, 1.68)	0.262
ACEI/ARB (n, %)		0 (0)	104 (83.87)	<0.001	84 (80.77)	20 (100.00)	0.041
Beta blockers (n, %)		0 (0)	84 (67.74)	<0.001	73 (70.19)	11 (55.00)	0.183
Diuretics (n, %)		0 (0)	117 (94.35)	<0.001	97 (93.27)	20 (100.00)	0.232
Digoxin (n, %)		0 (0)	70 (56.45)	<0.001	58 (55.77)	12 (60.00)	0.727

p* indicates healthy versus DCM, *$p^{\#}$* indicates no event group versus event group. ACEI, angiotensin converting enzyme inhibitor; ARB, angiotensin receptor blocker; BSA, body surface area; DCM, dilated cardiomyopathy; CKMB, creatine kinase myocardial band; DM, diabetes mellitus; HBP, high blood pressure; LBBB, left bundle branch block; NT-pro BNP, N-terminal pro-B-type natriuretic peptide; NYHA, New York Heart Association; TnI, troponin. Levels of CKMB <0.5 ug/L and TnI <0.017 ug/L were regarded as negative.

The DCM cohorts were divided into event group and no event group, which included 20 subjects (16.13%) and 104 subjects (83.87%), respectively. There were no significant differences in these two groups as related to age, sex, height, weight, or incidences of hypertension, diabetes mellitus, hyperlipidemia, and smoking (all *p* > 0.05). As for prevalence of LBBB, no patients with LBBB were recorded in the event group, while 19 patients with LBBB were detected in the no event group (*p* < 0.05). Patients in the event group were more likely to have NYHA functional classes >III (*p* < 0.05). NT-pro BNP levels were higher in the event group when compared to the no event group (7559 [3983, 12167] pg/mL vs. 1798 [887, 5145] pg/mL, *p* < 0.001). There were no statistical differences in levels of TnI or CKMB between the two subgroups (both *p* > 0.05). While the utilization of beta blockers, diuretics and digoxin were similar between the two subgroups, ACEI or ARB were more frequently utilized in the event group when compared to the no event group (*p* < 0.05).

### 3.2 Cardiac Function by CMR

In comparison to the healthy group, the DCM group exhibited several significant differences. These included higher heart rate (HR) and left ventricular mass index (LVMi), as well as lower CO, LVEF, RVEF, LA EF and right atrial (RA) EF (all *p* < 0.001). Furthermore, the DCM group had larger LV, RV EDD, biventricular volume, and biatrial volume (all *p* < 0.001) (Table 2).

**Table 2.** **Comparison of CMR parameters between healthy group, DCM group, no event group, and event group**.

	Healthy (n = 53)	DCM (n = 124)	p*	No event (n = 104)	Event (n = 20)	* $p^{\#}$ *
HR (1/min)	69.00 ± 11.00	83.00 ± 17.00	<0.001	80.00 ± 15.00	96.00 ± 22.00	0.005
CO (L/min)	6.04 ± 1.58	5.35 ± 2.02	0.028	5.37 ± 2.02	5.25 ± 2.04	0.814
LVMi (g/$m^{2}$)	47.93 ± 9.06	79.32 ± 19.41	<0.001	78.67 ± 19.42	82.72 ± 19.46	0.395
LVEF (%)	66.93 ± 7.07	21.98 ± 8.72	<0.001	23.43 ± 8.64	14.45 ± 4.09	<0.001
LV EDVi (mL/$m^{2}$)	73.15 ± 15.87	176.37 ± 48.57	<0.001	168.44 ± 43.25	217.63 ± 54.71	<0.001
LV ESVi (mL/$m^{2}$)	24.72 ± 7.17	142.84 ± 47.53	<0.001	133.36 ± 40.81	192.13 ± 50.48	<0.001
LV EDD (mm)	47.03 ± 4.44	72.69 ± 9.84	<0.001	71.51 ± 9.35	78.82 ± 10.29	0.002
RVEF (%)	51.26 ± 10.94	32.67 ± 16.47	<0.001	35.55 ± 15.24	17.66 ± 14.62	<0.001
RV EDVi (mL/$m^{2}$)	62.99 ± 13.82	95.17 ± 32.92	<0.001	89.85 ± 28.6	122.84 ± 40.26	<0.001
RV ESVi (mL/$m^{2}$)	31.78 ± 10.74	67.37 ± 36.51	<0.001	60.25 ± 30.31	104.4 ± 43.89	<0.001
RV EDD (mm)	27.6 ± 7.52	27.49 ± 10.9	0.939	26.55 ± 10.28	32.4 ± 12.89	0.027
LA EF (%)	57.16 ± 6.79	32.65 ± 14.24	<0.001	34.41 ± 14.6	23.52 ± 7.33	<0.001
LAVi min (mL/$m^{2}$)	19.74 ± 6.35	57.79 ± 35.5	<0.001	52.82 ± 29.32	83.66 ± 51.54	<0.001
LAVi max (mL/$m^{2}$)	40.44 ± 10.39	72.23 ± 34.15	<0.001	68.27 ± 27.84	92.83 ± 53.05	0.003
RA EF (%)	51 ± 12.3	44.95 ± 14.85	0.01	45.43 ± 14.45	42.46 ± 16.96	0.415
RAVi min (mL/$m^{2}$)	19.79 ± 7.88	29.38 ± 18.4	<0.001	27.38 ± 16.54	39.75 ± 23.95	0.005
RAVi max (mL/$m^{2}$)	33.37 ± 10.78	38.83 ± 17.6	0.013	37.01 ± 15.02	48.34 ± 25.89	0.071
LGE (%)	0 (0, 0)	6.70 (3.01, 12.87)	<0.001	6.13 (2.53, 12.61)	10.36 (6.11, 14.86)	0.056
LV GRS (%)	32.36 ± 6.07	8.06 ± 4.74	<0.001	8.77 ± 4.84	4.38 ± 1.09	<0.001
LV GCS (%)	–18.62 ± 2.13	–6.18 ± 2.85	<0.001	–6.65 ± 2.86	–3.72 ± 0.88	<0.001
LV GLS (%)	–17.57 ± 1.63	–6.41 ± 3.26	<0.001	–6.98 ± 3.24	–3.43 ± 0.96	<0.001
RV GRS (%)	26.46 ± 8.56	10.10 ± 7.17	<0.001	11.26 ± 7.09	4.08 ± 3.83	<0.001
RV GCS (%)	–17.09 ± 3.97	–5.19 ± 4.58	<0.001	–5.93 (–8.66, –3.75)	–1.79 (–3.77, 2.44)	<0.001
RV GLS (%)	–25.75 ± 4.79	–14.05 ± 11.43	<0.001	–16.96 (–20.68, –12.95)	–10.00 (–14.66, 3.30)	<0.001
LA GLS (%)	31.38 ± 11.44	10.61 ± 8.79	<0.001	11.71 ± 9.02	4.87 ± 4.16	<0.001
RA GLS (%)	36.07 ± 11.86	24.61 ± 15.3	<0.001	26.16 ± 15.27	16.52 ± 13.03	0.009

p* indicates healthy versus DCM, *$p^{\#}$* indicates no event group versus event group. CMR, cardiovascular magnetic resonance; CO, cardiac output; DCM, dilated cardiomyopathy; EDD, end-diastolic dimension; EDVi, end-diastolic volume index; EF, empty fraction; ESVi, end-systolic volume index; GCS, global circumferential strain; GLS, global longitudinal strain; GRS, global radial strain; HR, heart rate; LA, left atrial; LAVi max, maximum left atrial volume index; LAVi min, minimum left atrial volume index; LGE, late gadolinium enhancement; LVEF, left ventricular ejection fraction; LVMi, left ventricular mass index; RA, right atrial; RAVi max, maximum right atrial volume index; RAVi min, minimum right atrial volume index; RVEF, right ventricular ejection fraction; LV, left ventricular; RV, right ventricular.

HR was higher in the event group compared to the no event group in the DCM cohort (*p* < 0.001). CO and LVMi were not statistically different between the two subgroups (both *p* > 0.05). Lower LVEF and RVEF were measured in the event group as compared to the no event group (LV, 14.45 ± 4.09% vs. 23.43 ± 8.64%; RV, 17.66 ± 14.62% vs. 35.55 ± 15.24%; both *p* < 0.001). LV EDD, LV end-diastolic volume index (EDVi) and LV end-systolic volume index (ESVi) were significantly higher in the event group as compared to the no event group (LV EDD, 78.82 ± 10.29 mm vs. 71.51 ± 9.35 mm; LV EDVi, 217.63 ± 54.71 mL/$m^{2}$ vs. 168.44 ± 43.25 mL/$m^{2}$; LV ESVi, 192.13 ± 50.48 mL/$m^{2}$ vs. 133.36 ± 40.81 mL/$m^{2}$; all *p* < 0.001). RV EDD, RV EDVi, and RV ESVi were significantly increased in the event group as compared to the no event group (RV EDD, 32.40 ± 12.89 mm vs. 26.55 ± 10.28 mm; RV EDVi, 122.84 ± 40.26 mL/$m^{2}$ vs. 89.85 ± 28.60 mL/$m^{2}$; RV ESVi, 104.4 ± 43.89 mL/$m^{2}$ vs. 60.25 ± 30.31 mL/$m^{2}$; all *p* < 0.001). LA EF, minimum LA volume index (LAVi min) and maximum LA volume index LAVi max were reduced in the event group than no event group (LA EF, 23.52 ± 7.33% vs. 34.41 ± 14.6%; LAVi min, 83.66 ± 51.54 mL/$m^{2}$ vs. 52.82 ± 29.32 mL/$m^{2}$; LAVi max, 92.83 ± 53.05 mL/$m^{2}$ vs. 68.27 ± 27.84 mL/$m^{2}$; all *p* < 0.05). Minimum RA volume index (RAVi min) was higher in the event group as compared to the no event group (39.75 ± 23.95 mL/$m^{2}$ vs. 27.38 ± 16.54 mL/$m^{2}$; *p* < 0.05). RA EF and maximum RA volume index (RAVi max) were similar between the two subgroups (both *p* > 0.05).

### 3.3 Myocardial Strain and LGE

Biventricular GRS, GCS, and GLS were significantly impaired in the DCM group compared to the healthy group (all *p* < 0.05). GLS of LA and RA were also reduced in the DCM group relative to the healthy group (both *p* < 0.05). The extent of LGE was higher in the DCM group than the healthy group (*p* < 0.001) (Table 2).

**Fig. 3.**
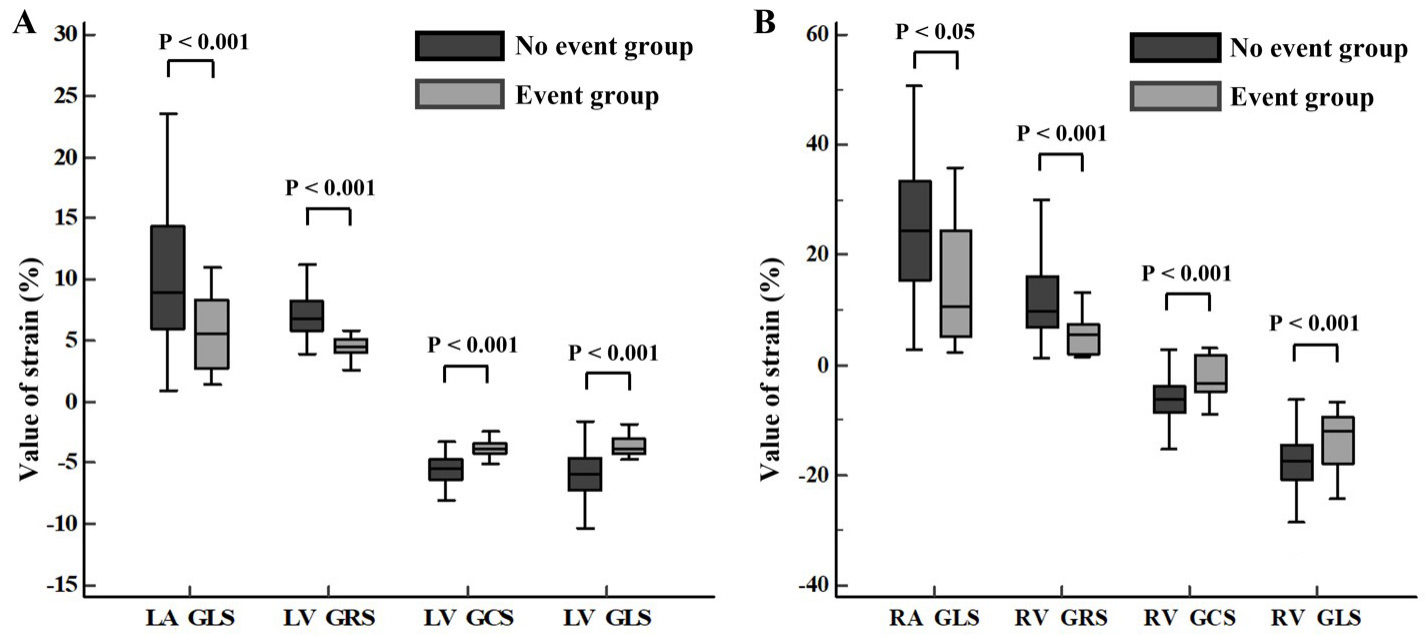
**Comparison of biventricular GRS, GCS, GLS, biatrial GLS between no event group and event group**. Values for the myocardial strain in the left (A) and right (B) side of the heart were statistically reduced in the event group relative to the no event group. GCS, global circumferential strain; GLS, global longitudinal strain; GRS, global radial strain; LA, left atrial; LV, left ventricular; RA, right atrial; RV, right ventricular.

Biventricular GRS was significantly lower in the event group relative to the no event group (LV, 4.38 ± 1.09% vs. 8.77 ± 4.84%; RV, 4.08 ± 3.83% vs. 11.26 ± 7.09%; both *p* < 0.001) (Fig. 3). Biventricular GCS was also impaired in the event group as compared to the no event group (LV, –3.72 ± 0.88% vs. –6.65 ± 2.86%; RV, –1.79 [–3.77, 2.44] % vs. –5.93 [–8.66, –3.75] %; both *p* < 0.001). Biventricular GLS was impaired in the event group as compared to the no event group (LV, –3.43 ± 0.96% vs. –6.98 ± 3.24%; RV, –10.00 [–14.66, 3.30] % vs. –16.96 [–20.68, –12.95] %; both *p* < 0.001). Biatrial GLS was statistically reduced in the event group as compared to the no event group (LA, 4.87 ± 4.16% vs. 11.71 ± 9.02%; RA, 16.52 ± 13.03% vs. 26.16 ± 15.27%; both *p* < 0.05). There were no statistical differences regarding the extent of LGE between the event group and the no event group (*p* > 0.05).

### 3.4 Clinical Outcomes

Over a median follow-up period of 362 days (IQR: 234 to 500 days), MACE occurred in 20 patients (16.13%) (all-cause death, n = 5; heart transplantation, n = 1; life-threatening arrhythmia, n = 6; hospitalizations due to heart failure, n = 8). The univariate Cox regression analysis revealed that Ln (NT-pro BNP), LGE, HR, LV EDD, RV EDD, LV EF, LV EDVi, LV ESVi, RV EF, RV EDVi, RV ESVi, LA EF, LAVi min, LAVi max, LV GRS, LV GCS, LV GLS, RV GRS, RV GCS, RV GLS, LA GLS, RA GLS were all significant predictors of MACE (all *p* < 0.05). Based on the univariate analysis and the sample size in our study, a multivariate Cox regression model including eight parameters (LVEF, RVEF, LV GRS, LV GCS, LV GLS, RV GRS, RV GCS, RV GLS) was assessed, and we found that LV GLS continued to predict MACE (1.788 [1.048, 3.049]; *p* < 0.05) (Table 3).

**Table 3.** **Univariate and multivariate analysis for predicting MACEs**.

	Univariate		Multivariate	
	HR (95% CI)	*p*	HR (95% CI)	*p*
Ln (NT-pro BNP)	1.907 (1.252, 2.904)	0.003		
LGE (%)	1.051 (1.011, 1.094)	0.013		
Heart rate (1/min)	1.021 (1.002, 1.041)	0.033		
LV EDD (mm)	1.070 (1.030, 1.110)	<0.001		
RV EDD (mm)	1.054 (1.015, 1.096)	0.007		
LVEF (%)	0.850 (0.774, 0.932)	0.001	1.000 (0.873, 1.145)	1.000
LV EDVi (g/$m^{2}$)	1.014 (1.007, 1.021)	<0.001		
LV ESVi (g/$m^{2}$)	1.016 (1.009, 1.023)	<0.001		
RVEF (%)	0.928 (0.894, 0.964)	<0.001	0.948 (0.895, 1.004)	0.067
RV EDVi (g/$m^{2}$)	1.023 (1.011, 1.035)	<0.001		
RV ESVi (g/$m^{2}$)	1.028 (1.016, 1.040)	<0.001		
LA EF (%)	0.930 (0.891, 0.972)	0.001		
LAVi min (g/$m^{2}$)	1.013 (1.008, 1.024)	<0.001		
LAVi max (g/$m^{2}$)	1.014 (1.005, 1.022)	0.002		
LV GRS (%)	0.616 (0.490, 0.774)	<0.001	0.060 (0.002, 2.387)	0.134
LV GCS (%)	1.828 (1.370, 2.440)	<0.001	0.037 (0.000, 3.598)	0.158
LV GLS (%)	1.854 (1.370, 2.510)	<0.001	1.788 (1.048, 3.049)	0.033
RV GRS (%)	0.864 (0.792, 0.941)	0.001	1.232 (0.952, 1.595)	0.113
RV GCS (%)	1.182 (1.070, 1.305)	0.001	1.067 (0.815, 1.397)	0.637
RV GLS (%)	1.043 (1.017, 1.071)	0.001	1.013 (0.974, 1.053)	0.512
LA GLS (%)	0.953 (0.912, 0.995)	0.030		
RA GLS (%)	0.961 (0.928, 0.995)	0.027		

CI, confidence interval; EDD, end-diastolic diameter; EDVi, end-diastolic volume index; EF, empty fraction; ESVi, end-systolic volume index; GCS, global circumferential strain; GLS, global longitudinal strain; GRS, global radial strain; HR, hazard ratio; LA, left atrial; LAVi max, maximum left atrial volume index; LAVi min, minimum left atrial volume index; LGE, late gadolinium enhancement; LV, left ventricular; LVEF, left ventricular ejection fraction; MACEs, major adverse cardiac events; NT-pro BNP, N-terminal pro-B-type natriuretic peptide; RV, right ventricular; RVEF, right ventricular ejection fraction.

**Fig. 4.**
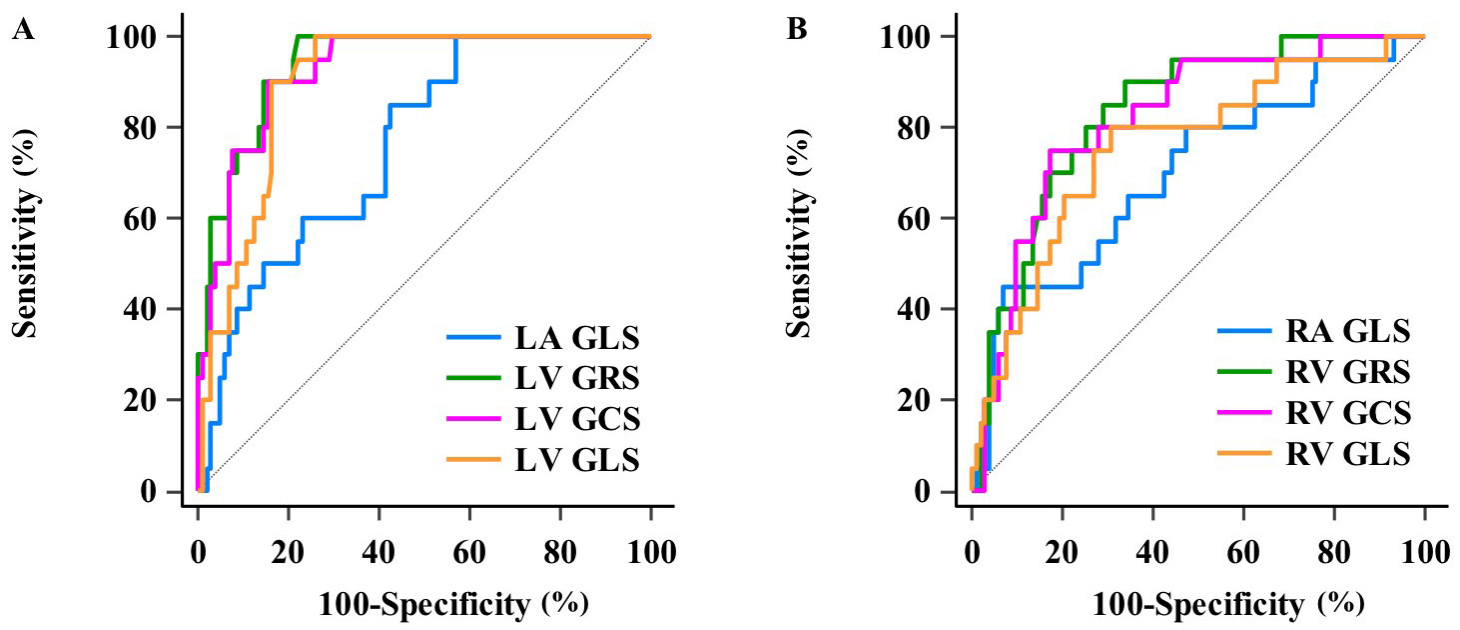
**ROC showed significant prognostic values of biatrial GLS, biventricular GRS, GCS, GLS in predicting MACEs**. The AUCs, sensitivity, specificity of the left (A) and right (B) side of the heart were displayed. GCS, global circumferential strain; GLS, global longitudinal strain; GRS, global radial strain; LA, left atrial; LV, left ventricular; MACE, major adverse cardiac event; RA, right atrial; ROC, receiver-operating characteristic; AUCs, area under the curves; RV, right ventricular.

**Fig. 5.**
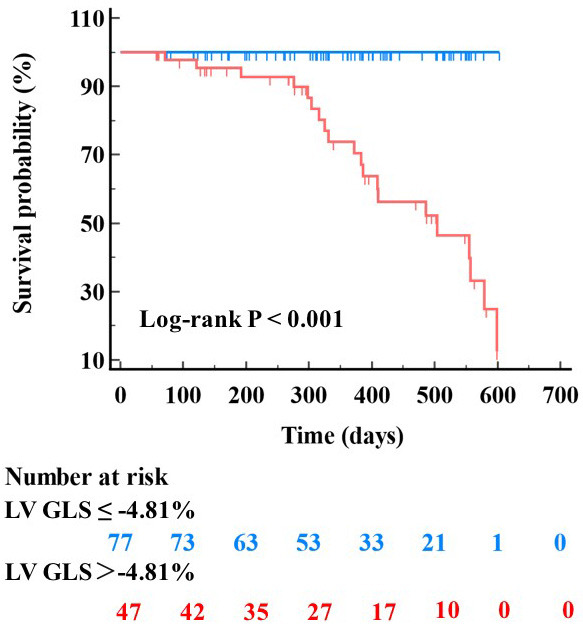
**Kaplan-Meier survival analyses for the prediction of MACEs**. An optimal cut-off value for LV GLS was identified at -4.81%, patients with a LV GLS >–4.81% exhibited a significantly higher rate of MACEs. GLS, global longitudinal strain; LV, left ventricular; MACE, major adverse cardiac event.

The ROC analysis revealed that the AUCs for the LA and RA GLS used to predict MACEs were 0.762 and 0.701 respectively (both *p* < 0.05). The following parameters demonstrated significant prognostic value in predicting MACEs: LV GRS (AUC: 0.939, sensitivity: 100.00%, specificity: 77.88%), LV GCS (AUC: 0.926, sensitivity: 90.00%, specificity: 84.62%), LV GLS (AUC: 0.900, sensitivity: 100.00%, specificity: 74.04%), and RV GRS (AUC: 0.834, sensitivity: 90.00%, specificity: 66.35%), RV GCS (AUC: 0.819, sensitivity: 75.00%, specificity: 82.69%), RV GLS (AUC: 0.759, sensitivity: 80.00%, specificity: 69.23%) showed significant prognostic value in predicting MACE (all *p* < 0.05) (Fig. 4). Using the Youden index, an optimal cut-off value for LV GLS was identified at –4.81% to categorize patients with high risk for events. According to the results in the ROC analysis, patients with a LV GLS >–4.81% exhibited a significantly higher rate of MACEs in Kaplan-Meier analysis (Log-rank *p* < 0.001) (Fig. 5).

### 3.5 Correlations

In DCM patients, LV GLS showed strong negative correlations with LVEF and LA EF (both *p* < 0.001). LV GLS showed moderate positive correlations with LV ESVi and LAVi min (both *p* < 0.001). Values for LV GLS correlated weakly with LVMi, LAVi max and LV EDVi (all *p* < 0.001). Furthermore, LA GLS presented strong negative correlations with LAVi min and LAVi max (both *p* < 0.001). LA GLS presented a strong positive correlation with LA EF (*p* < 0.001). Finally, LA GLS correlated weakly with LV EDVi, LV ESVi and LVEF (all *p* <0.001) (Table 4).

**Table 4.** **Correlations of LA and LV GLS with functional and structural parameters in DCM patients**.

	LA GLS		LV GLS	
Parameter	r value	*p* value	r value	*p* value
LV EDVi	–0.439	<0.001	0.447	<0.001
LV ESVi	–0.455	<0.001	0.537	<0.001
LAVi min	–0.720	<0.001	0.508	<0.001
LAVi max	–0.616	<0.001	0.384	<0.001
LVEF	0.434	<0.001	–0.691	<0.001
LA EF	0.690	<0.001	–0.618	<0.001
LVMi	–0.169	0.06	0.346	<0.001

EDVi, end-diastolic volume index; EF, empty fraction; ESVi, end-systolic volume index; GLS, global longitudinal strain; LA, left atrial; LAVi max, maximum left atrial volume index; LAVi min, minimum left atrial volume index; LV, left ventricular; LVEF, left ventricular ejection fraction; LVMi, left ventricular mass index; DCM, dilated cardiomyopathy.

### 3.6 Observer Variability

**Fig. 6.**
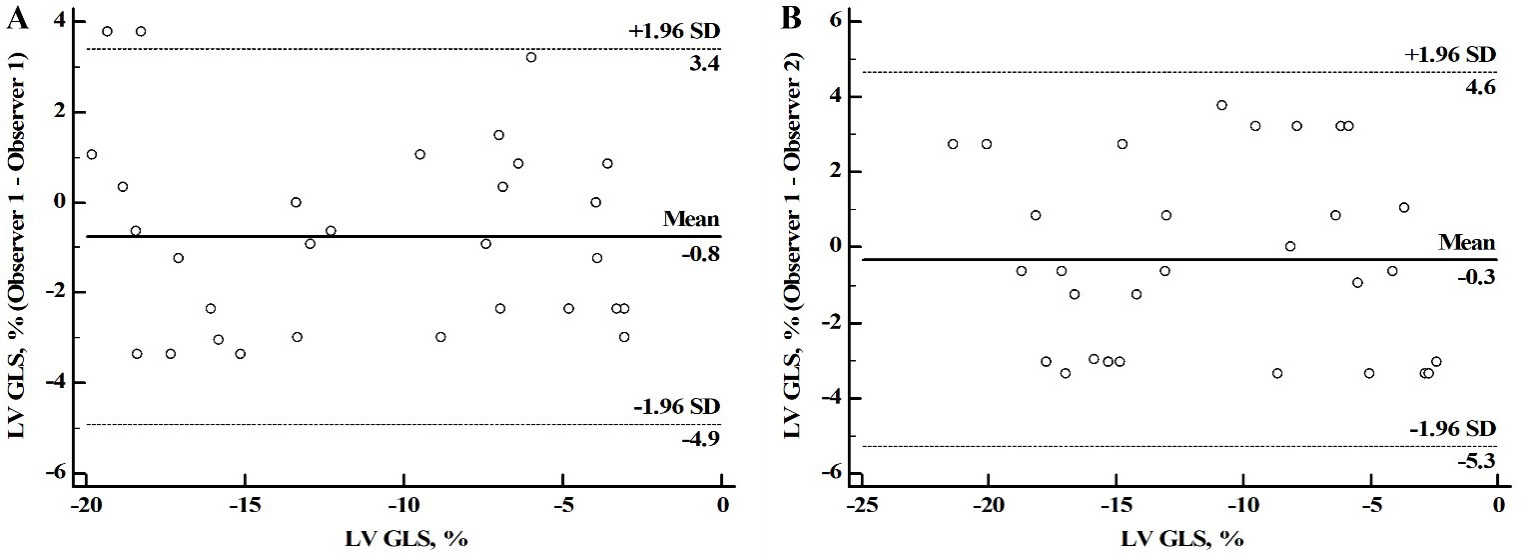
**The high reproducibility of strain measurement in DCM and healthy groups**. The intraobserver variability (A) and interobserver variability (B) of LV GLS in the two groups. DCM, dilated cardiomyopathy; GLS, global longitudinal strain; LV, left ventricular.

Measurement of cardiac strain parameters revealed solid reproducibility in healthy volunteers and DCM patients. The ICC for the intraobserver variability was found to be 0.933 (95% CI: 0.859–0.968) for LV GLS. Similarly, the ICC for the interobserver variability for LV GLS also had a high level of agreement with a value of 0.913 (95% CI: 0.827–0.958) (Fig. 6). These results indicate the reliability and consistency of the strain measurements in this patient population.

## 4. Discussion

In this study, we employed FT and fast long-axis methods evaluate deformation characteristics of the ventricle and atrium in patients diagnosed with DCM. Our study yielded several important findings: The DCM group exhibited impaired biventricular GRS, GCS, GLS, and biatrial GLS in comparison to the healthy group. Furthermore, the event group demonstrated significantly reduced biventricular GRS, GCS, GLS, and biatrial GLS compared to the no event group. While the extent of LGE was higher in the DCM group compared to the healthy group, no statistical differences were observed between the event group and the no event group. Notably, LV GLS demonstrated significant and independent prognostic value outperforming other CMR parameters in predicting MACEs over a median follow-up of 362 days.

The reduction of biventricular GRS, GCS, GLS, and biatrial GLS were observed in the DCM group, which was consistent with a previous study about myocardial deformation characteristics [26]. In the early stage of DCM, cardiac function can be compensated by the Frank-Starling law, which enables an increase in myocardial contractility and helps to regulate reduced stroke volume [27]. However, in DCM patients who reached the end stage of disease, decreased cardiac function is consistently accompanied with enlargement of the ventricular chamber and decreased ventricular compliance [28].

The reduction in LV stroke volume contributes to an increase in preload, consequently leading to LA dysfunction [11, 29]. The interplay between LA and LV, pulmonary vascular pressure increases further contributes to RV dysfunction [9]. These mechanisms align with the findings of our study. The treatment methods for DCM include medication, cardioverter defibrillator implantation, resynchronization therapy, and heart transplantation. Early identification of disease characteristics and ultimate progression is crucial for guiding appropriate treatment.

LV GLS remains as an independent predicting parameter for other cardiac pathologies, including myocarditis, myocardial infarction, and even heart transplantation [30, 31, 32]. GLS refers to systolic shortening of the cardiac chamber in long-axis direction, which can be used to evaluate the motion ability of the ventricle in the cardiac cycle [33]. Raafs *et al*. [34] provided evidence that speckle tracking echocardiographic LV GLS emerged as an independent and incremental predictor of adverse outcome other than LVEF in patients with DCM. LV GLS has been suggested to be routinely measured for DCM prognosis assessment. Another study found RV GLS to be independently associated with MACEs independent of the of interaction between LA and RA [22], an event that is contradictory to our finding. We only found LV GLS to be an independent prognostic parameter that predicted MACE. There were also strong correlations between atrial and ventricular strain and CMR functional parameters, including LVEF and LA EF. Therefore, LA and RA-related parameters should not be underestimated, and all four chambers should be coordinated to assess overall pathological changes. The recognition of myocardial dysfunction in DCM patients is crucial for risk stratification and prediction of prognosis [35].

LGE was superior and independent from LVEF in predicting arrhythmic events, although LVEF was considered the main factor for selecting candidates for primary prevention with an implantable cardioverter-defibrillators [36]. Researchers have demonstrated that myocardial fibrosis is associated with increasing risk of ventricular arrhythmias [37]. The presence of LGE offers a powerful value in prognosis evaluation in non-ischemic cardiomyopathy [1, 2, 38]. In our study, LGE was only shown to be an important predictor of adverse outcomes under univariate Cox regression analysis. We believe this disparity might be explained by two points, (1) the relatively shorter follow-up period may account for the difference, (2) the total DCM cohort tended to be in the late stage of disease in our study population.

LA function is closely intertwined with LV function and impaired LV function is typically accompanied by a decrease in LA performance. The involvement of LA in regulating LV is divided into three phases: (1) reservoir phase, which involves the collection of pulmonary venous return during LV contraction; (2) conduit phase, where blood is passed to LV during early diastole; and (3) booster pump phase, which entails the augmentation of LV filling by atrial contraction during late diastole [39]. LA GLS has been extensively studied in various conditions including heart failure, atrial fibrillation, and myocardial fibrosis using multiple CMR techniques [40, 41]. Previous studies have examined LA reservoir strain, conduit strain, and booster strain to explore the influences of LA in different diseases [9, 18, 19]. In our study patients with DCM experienced significant impairments in both LA and LV function, making it challenging to differentiate LA function into these three distinct phases. Therefore, we solely considered LA GLS as a potential parameter that might influence the study results.

There were several limitations to this study. First, it was a single-center retrospective study including a small number of patients. Prospective research involving a larger study population is needed to validate our results. Second, by the study completion date, 8 parameters were included in the multivariate Cox regression analysis. For a more convincing statistical analysis, a larger study sample would be more appropriate. Third, the median follow-up period was 362 days (IQR: 234 to 500 days) and only 20 MACEs were recorded. A longer follow-up period should aid in the search for a prognosis response. Fourth, the majority of participants in our study were DCM patients with significantly reduced LVEF. It is important to note that our study specifically focused on DCM patients with severe systolic dysfunction, and therefore, the findings may not be directly applicable to those with mild to moderate impairment. Conducting a multi-center research study would be essential to mitigate this potential bias and provide more comprehensive insights. Finally, our study solely evaluated LGE through the quantification of its volumetric proportion of the total LV myocardium. The pattern or distribution of LGE might offer additional novel viewpoints. Multiple LGE-related parameters have the potential to provide insights leading to improved clinical outcomes.

## 5. Conclusions

By using FT and fast long-axis method derived from CMR, we found that biventricular GRS, GCS, GLS, and biatrial GLS were significantly impaired in the event group relative to the no event group in DCM. LV GLS was independently associated with MACE in DCM patients. Comprehensive CMR examination should be systematically performed, in order to understand disease characteristics, as well as improve the risk stratification and therapeutic management for patients with DCM.

## Data Availability

The datasets supporting the conclusions of the current study are available from the corresponding author on reasonable request.
